# Structured effects of exercise on circulating brain-derived neurotrophic factor (BDNF) in postmenopausal women: a meta-analysis

**DOI:** 10.3389/fphys.2026.1953636

**Published:** 2026-10-22

**Authors:** Ying Zhou, Jianhui Chai, Jin Wang, Jian Wang

**Affiliations:** 1 Faculty of Sports Science, Ningbo University, Ningbo, China; 2 Physical Education Teaching and Research Group, Ningbo Foreign Affairs School, Ningbo, China; 3 Department of Physical Education, Ningbo University of Technology, Ningbo, China

**Keywords:** BDNF, brain−derived neurotrophic factor, menopausal women, meta−analysis, postmenopausal women, resistance training

## Abstract

**Objective:**

To systematically evaluate the effect of structured exercise interventions on circulating brain-derived neurotrophic factor (BDNF) levels among postmenopausal women, and to clarify the moderating effects of intervention modality, population characteristics, training duration, exercise intensity and energy-supply pattern on intervention outcomes, so as to provide evidence-based references for precise exercise interventions targeting brain health in postmenopausal women.

**Methods:**

Following the Preferred Reporting Items for Systematic Reviews and Meta-Analyses (PRISMA) statement, electronic databases including PubMed, Web of Science, EBSCO and CNKI were searched for original studies regarding the effects of various exercise interventions on BDNF in postmenopausal women, from database inception to August 1, 2026. The Cochrane Risk-of-Bias Tool was applied for randomized studies. For quasi-randomized and non-randomized intervention studies, we explicitly acknowledge limitations of this tool and interpret methodological assessments cautiously; studies with insufficient randomization reporting were rated as unclear risk of bias. Meta-analysis was performed using Review Manager 5.4 software. Effect sizes were presented as standardized mean difference (SMD) with 95% confidence interval (95% CI). Subgroup analysis, sensitivity analysis and publication-bias evaluation were also conducted.

**Results:**

Twelve studies involving 351 participants were finally included, with 173 subjects in the intervention group and 178 subjects in the control group. All included trials adopted chronic long-term structured exercise interventions without acute single-bout exercise protocols. Pooled meta-analysis demonstrated that structured exercise interventions significantly elevated BDNF levels in postmenopausal women compared with control conditions [SMD = 0.64, 95% CI (0.11, 1.17), P = 0.02], with substantial between-study heterogeneity (I² = 81%, P < 0.0001). Subgroup analyses (only subgroups with ≥3 studies included for quantitative synthesis) indicated that intervention modality, population characteristics, training duration and energy-supply pattern were potential main sources of heterogeneity. Statistically-significant maximum effect sizes were observed in the resistance training subgroup [SMD = 1.07, 95% CI (0.12, 2.01), P = 0.03], healthy older/postmenopausal-women subgroup [SMD = 0.30, 95% CI (-0.05, 0.65), P = 0.009], anaerobic-energy-supply-predominant subgroup [SMD = 1.07, 95% CI (0.12, 2.01), P = 0.03]. Some potential moderators could not be quantitatively evaluated because corresponding subgroups contained fewer than three primary studies, and those comparisons are only discussed as exploratory clues rather than robust conclusions. Sensitivity analysis revealed no substantial shift in pooled effect direction after sequential exclusion of individual studies. Funnel-plot inspection and Egger’s test detected no statistical evidence of publication asymmetry; nevertheless, these assessments were limited by the small number of included studies and cannot rule out publication bias definitively.

**Conclusion:**

Structured exercise interventions can significantly increase peripheral BDNF levels in postmenopausal women, which suggests beneficial impacts on neuroplasticity and brain health for this population. Chronic resistance-based exercise regimens dominated by anaerobic energy-supply exert superior regulatory effects among healthy postmenopausal women.

**Systematic review registration:**

https://www.crd.york.ac.uk/PROSPERO/view/CRD420261468069, identifier CRD420261468069.

## 1 Introduction

With the accelerating global population ageing, health-related issues among postmenopausal women have become an important topic in the fields of public health and sports medicine. Menopause generally occurs at the age of 45–55 years and represents a critical transitional phase in the female life cycle. Ovarian function declines after menopause, accompanied by reduced circulating oestrogen levels, which trigger multi-system alterations involving neuroendocrine, metabolic, musculoskeletal and psychological functions, and further increase risks of osteoporosis, cardiometabolic abnormalities, sleep disturbances, mood disorders and cognitive decline. The World Health Organization reported that women aged 50 years and older accounted for 26 % of the total global female and girl population in 2021, representing a notable increase compared with a decade ago (World Health Organization, 2024). Therefore, exploring safe, economical and readily scalable non-pharmacological interventions to improve physical-mental health and neural function among postmenopausal women carries important practical implications.

Brain-derived neurotrophic factor (BDNF) is a vital member of the neurotrophin family. It is predominantly expressed in the central nervous system (CNS), with high concentrations detected in brain regions closely linked to learning, memory and emotional regulation, such as the hippocampus, cerebral cortex and forebrain. BDNF exerts biological functions mainly via binding to tropomyosin receptor kinase B (TrkB), participating in neuronal survival, differentiation, axonal outgrowth, synaptogenesis and modulation of synaptic plasticity; accordingly, it serves as an essential molecular foundation for maintaining the structural and functional stability of the nervous system (Leal et al., 2017). Accumulating evidence demonstrates that reduced BDNF levels are strongly associated with ageing, cognitive impairment, depressive disorders, Alzheimer’s disease and metabolic disturbances. Peripheral circulating BDNF is regarded as a surrogate biomarker; measured changes in blood BDNF do not directly demonstrate alterations within the central nervous system. Thus, BDNF is widely recognised as a key peripheral biomarker reflecting putative neuroplasticity and neuroprotective status (Miranda et al., 2019; Notaras and van den Buuse, 2020).

BDNF bears distinctive research value for postmenopausal women. Postmenopausal oestrogen decline not only compromises vascular, skeletal and metabolic health but may also modulate neuroplasticity and cognitive function by regulating BDNF and its downstream signalling pathways. Previous studies have indicated reciprocal interactions between oestrogen and BDNF: oestrogen participates in the regulation of BDNF expression, whereas altered BDNF signalling is potentially linked to postmenopausal cognitive decline, mood disturbance and attenuated neuroprotective capacity (Numakawa et al., 2010). Consequently, optimising BDNF levels constitutes a promising strategy for promoting brain health and mitigating cognitive deterioration in postmenopausal women.

Structured exercise acts as a critical non-pharmacological approach for modulating BDNF concentrations. Existing investigations have illustrated that acute single-bout exercise can transiently elevate circulating BDNF levels, yet long-term regular chronic exercise may modify basal resting BDNF concentrations and cognitive performance through sustained adaptive physiological adaptations, which possess higher clinical persuasive value (Dinoff et al., 2016). Exercise-driven BDNF regulation may involve enhanced cerebral blood flow, improved insulin sensitivity, suppressed chronic inflammation, stimulated secretion of skeletal-muscle-derived myokines, and modulation of neuroplasticity through the muscle-brain axis. Previous meta-analyses have shown that exercise training generally increases peripheral resting BDNF levels; nevertheless, effect magnitudes differ across exercise modalities. Ample evidence supports aerobic exercise, while the differential effects of diverse exercise modalities on BDNF remain controversial (Dinoff et al., 2016).

Multiple types of structured exercise are core components of exercise prescriptions for older adults. It improves muscular strength, muscle mass, physical function, bone mineral density and metabolic health. The World Health Organization recommends that older adults perform muscle-strengthening activities of moderate-to-vigorous intensity on no fewer than two days per week to preserve physical function and foster healthy ageing (World Health Organization, 2020) For postmenopausal women, various structured exercise interventions alleviate sarcopenia, bone loss and functional deterioration. Furthermore, it may alter BDNF levels via muscle contraction-derived stimulation, inflammatory modulation, metabolic improvement and neurotrophin release (Fragala et al., 2019; González-Gálvez et al., 2024). However, original studies specifically investigating exercise-induced BDNF changes in postmenopausal women remain limited, and reported outcomes are inconsistent. Studies focusing purely on single exercise modality among clearly-defined postmenopausal women are particularly scarce. Therefore, this meta-analysis broadened the inclusion scope to multiple forms of chronic structured exercise interventions and excluded acute single-bout exercise trials. To avoid unstable pooled estimates, we only performed quantitative subgroup synthesis for strata containing at least three independent studies; small-sample subgroups with less than three studies were not pooled and are only mentioned descriptively. Subgroup analyses further explored effect differences across eligible exercise-related factors.

Most prior meta-analyses pooled participants with heterogeneous sex, age, health status and exercise modalities, making it difficult to synthesize exercise-related BDNF responses specifically among postmenopausal women. In addition, substantial methodological heterogeneity exists across available studies with respect to intervention protocols, training duration, exercise intensity, participant health conditions, BDNF specimen types and detection assays, which may contribute to divergent findings. Several trials reported significant BDNF elevation following exercise intervention, whereas others detected no measurable changes. Therefore, systematic integration of current evidence and exploration of potential exploratory sources of heterogeneity are warranted. Accordingly, the present meta-analysis was performed to systematically evaluate the influence of chronic structured exercise interventions on BDNF levels in postmenopausal women. Subgroup analyses were conducted based on intervention modality, intervention duration, exercise intensity, population characteristics and energy-supply pattern. Given limited sample size, formal interaction tests for subgroup differences could not be completed; observed between-subgroup disparities in statistical significance cannot be interpreted as definitive proof of moderating effects. Findings from this work may provide evidence-based support for clarifying the role of exercise in neuroprotection and cognitive health for postmenopausal women, and offer references for exercise prescription formulation, community health interventions and the design of high-quality randomised controlled trials targeting this population.

## 2 Materials and methods

### 2.1 Search strategy

To improve the transparency and reproducibility of literature search and screening procedures, the present study conducted systematic retrieval strictly following the Preferred Reporting Items for Systematic Reviews and Meta-Analyses (PRISMA) statement(CRD420261468069) (Schmidt et al., 2019). Two researchers independently performed literature searching, screening and cross-verification, and any discrepancies were resolved through discussion. Databases including PubMed, Web of Science, EBSCO and CNKI were searched from database inception to August 1, 2026. Search terms were developed around three core themes: structured exercise intervention, postmenopausal women, and brain-derived neurotrophic factor (BDNF). The English search string was: (“exercise” OR “physical training” OR “resistance training” OR “aerobic exercise” OR “strength training” OR “whole-body vibration”) AND (“menopause” OR “postmenopausal” OR “menopausal women” OR “older women”) AND (“brain-derived neurotrophic factor” OR “BDNF”). Chinese search terms included resistance training, strength training, resistance exercise, elastic-band training, menopausal women, postmenopausal women, older women, brain-derived neurotrophic factor, and BDNF. Both subject terms and free-text terms were applied for retrieval. References of the included studies were also hand-searched to identify as many relevant publications as possible. After initial retrieval, duplicate removal, title-and-abstract screening, and full-text eligibility assessment, 12 studies meeting the inclusion criteria were ultimately enrolled, and acute single-bout exercise studies were excluded. The detailed literature-screening process is shown in the PRISMA flow diagram (**Figure 1**).

**Figure 1:**
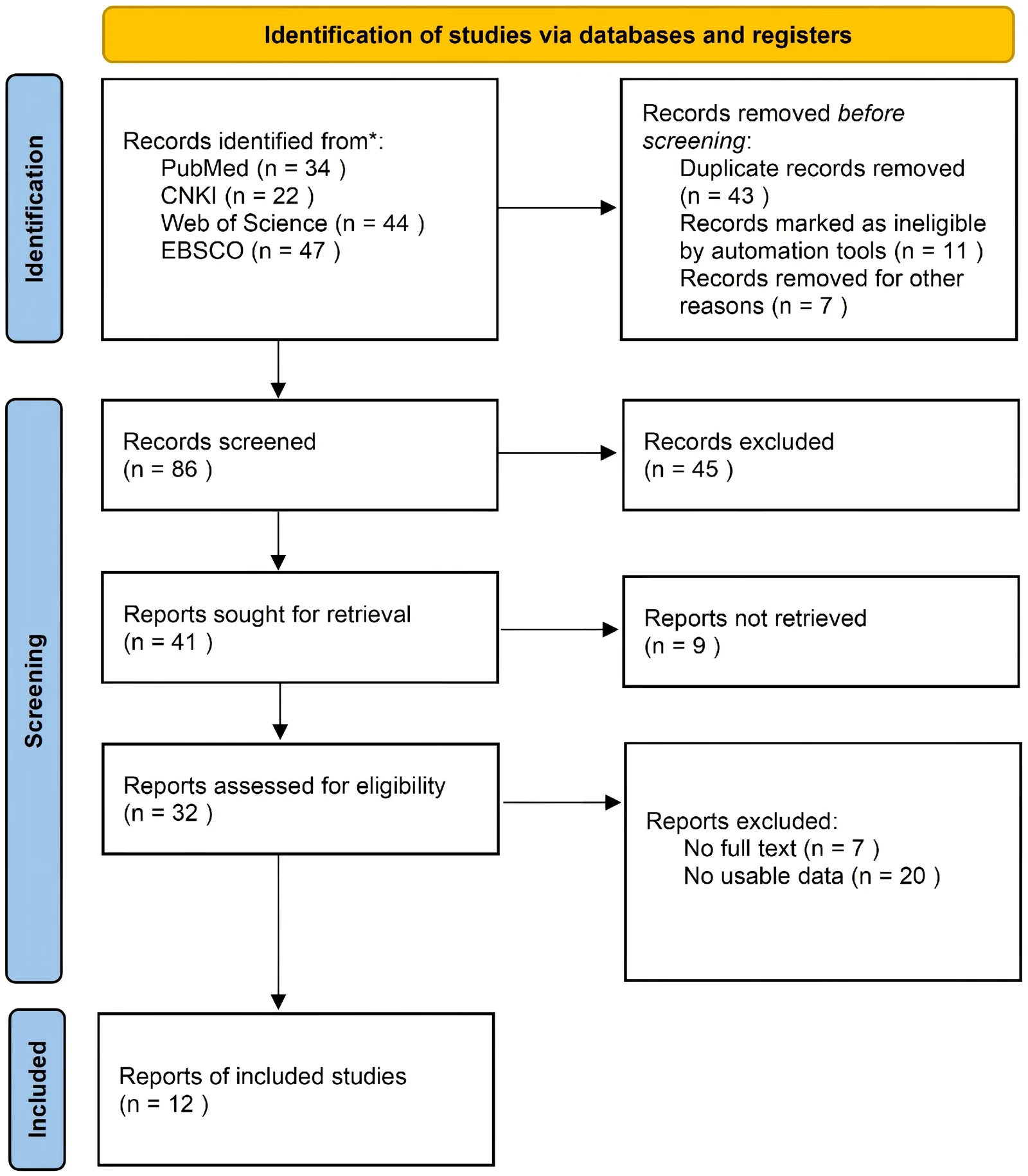
Flow diagram of literature selection.

### 2.2 Inclusion and exclusion criteria

To ensure consistency and reduce selection bias, eligibility criteria for this meta-analysis were explicitly formulated according to the PICOS principle (Amir-Behghadami and Janati, 2020). All components are clearly defined as follows:

#### 2.2.1 Inclusion criteria

##### 2.2.1.1 Participants

Postmenopausal women were included. Subjects could be healthy women, women with obesity, pre-frail women, or older women with stable chronic diseases. Menopausal status must be explicitly reported with clear diagnostic definitions for postmenopausal status. Age alone was not accepted as sufficient evidence to confirm postmenopausal status. For studies mixing perimenopausal, menopausal and postmenopausal participants, studies were only retained if separate quantitative data for postmenopausal women could be extracted.

##### 2.2.1.2 Intervention

The experimental group received any form of chronic multi-week structured exercise intervention, including resistance-training-dominant regimens, combined exercise training, vibration-based training. Resistancetraining modalities included traditional resistance training, elasticband training, aquatic training, circuit resistance training, blood-flow-restriction training, and combined with other exercise modalities. Acute single-bout exercise protocols were excluded from eligibility.

##### 2.2.1.3 Comparator

Control conditions included no-exercise control, usual-lifestyle control, stretching training, sham intervention, placebo intervention, or other non-resistance/low-intensity comparative interventions. For studies containing multiple intervention arms, resistance-training-related experimental groups and their matched control groups were preferentially extracted. Where multiple experimental arms shared a single control group, the control-group sample was proportionally split across relevant comparisons to avoid double-counting control participants. Original sample size for each study arm, selected arms for primary meta-analysis, splitting procedure for shared controls, and final comparison sets are documented within the results section.

##### 2.2.1.4 Outcome measures

Studies had to report data related to brain-derived neurotrophic factor (BDNF), including serum, plasma or peripheral-blood BDNF concentrations. Acceptable data formats included mean ± standard deviation, P-values, or quantitative data extractable from figures or tables. Only chronic/resting-state BDNF measured after multi-week training was eligible for inclusion; BDNF outcomes collected immediately after a single acute exercise bout were excluded.

##### 2.2.1.5 Study design

Published randomized controlled trials, quasi-randomized controlled trials, or clinical intervention studies were eligible. Full-text articles were required, with clear reporting of intervention protocols, sample sizes, and pre-post or between-group BDNF outcomes. The specific study design (RCT/quasi-RCT/non-randomized clinical intervention study) for each included report is specified in **Table 1** (newly-added “Study design” column). In the results section, we explicitly distinguish RCTs from quasi-randomized or non-RCT clinical intervention studies, rather than uniformly labelling all included studies as RCTs.

**Table 1:** Characteristics of studies included in the systematic review and meta-analysis.

Study	Country	Age (years)	N (EG/CG)	Postmenopausal status	Intervention		Plan (Period)	Outcome extracted
					EG	CG		
Hosseini et al., 2026	Iran	66 ± 4.36	postmenopausal women 12/12	≥12 months without menses	Water-based resistance training	Inactive control	3 times/week; 3 circuits in weeks 1-4, 6 circuits in weeks 5-8, and 8 circuits in weeks 9-12 (12 weeks)	BDNF ↑
Jóźwiak et al., 2025	Poland	51.37 ± 4.71	postmenopausal women 21/16	≥12 months without menses	Exercise	Exercise +16:8 time-restricted eating	2 times/week, 55 min/session; 3 circuits; resistance intensity progressed from 50% to 60% to 70% 1RM (12 weeks)	NS
Huang et al., 2025	Taiwan	65-75	postmenopausal women 14/14	≥1 year since last	Eccentric resistance training	Stretching	2times/week supervised + home-based training 7 eccentric exercises (8 weeks)	BDNF ↓
de Souza et al., 2024	Brazil	60-80	postmenopausal women n=42	≥1 year since last	IMT+WBV	Sham IMT + WBV/sham intervention	(12 weeks)	NS
Ghodrati et al., 2023	Iran	NR	postmenopausal women 12/9	≥1 year since last	Aerobic +resistance	No training	3 times/week (12 weeks)	BDNF ↓
Daďová et al., 2022	Czech	70.8 ± 3.6	postmenopausal women 27/25	≥1 year since last	Exercise + Calanus oil	Exercise + placebo	3 times/week; 1 Nordic walking session + 2 low-resistance circuit training sessions per week, about 1 h/session (16 weeks)	BDNF ↑
Asghari et al., 2021	Iran	50-70	postmenopausal women 13/12	≥1 year since last	MHRCT + placebo/MHRCT + ursolic acid	Control	Moderate- to high-intensity resistance circuit training; detailed sets/repetitions not reported (8 weeks)	BDNF ↑
Kargaran et al., 2021	Iran	62.9 ± 3.1	postmenopausal women 24/24	≥1 year since last	Dual-task training	Dual-task training + BFR/control	3 times/week, 20 min/session; treadmill at 45% HRR; BFR pressure 50%, increased by 10% every 2 weeks (8 weeks)	BDNF ↑
Coelho-Júnior et al., 2020	Brazil	66.7 ± 4.6	postmenopausal women 10/10	≥12 months without menses	Resistance training/power resistance training	Control	2 times/week; TRT: 3 sets × 8–10 repetitions, RPE 5-6; PTRT: 3 sets × 8–10 repetitions, RPE 3 (22 weeks)	BDNF ↑
Urzi et al., 2019	Slovenia	84	postmenopausal women 11/9	≥12 months without menses	Elastic resistance training	Control	Moderate-intensity ERT (12 weeks)	BDNF ↑
Coelho et al., 2012	Brazil	NR	postmenopausal women 48/20	≥12 months without menses	Physical therapy intervention	NR	8 weeks	BDNF ↑
Tsai and Chang, 2026	Taiwan	NR	postmenopausal women 22/22		Aerobic exercise	Aerobic exercise + WBV	16 weeks	BDNF ↑

EG, experimental group; CG, control group; BDNF, brain-derived neurotrophic factor; NS, no statistical significance; NR, not reported; ↑, significant increase; ↓, significant decrease; 1RM, one-repetition maximum; HIIAE, high-intensity interval aerobic exercise; WBV, whole-body vibration; IMT, inspiratory muscle training; MHRCT, moderate- to high-intensity resistance circuit training; BFR, blood flow restriction; HRR, heart rate reserve; TRT, traditional resistance training; PTRT, power resistance training; RPE, rating of perceived exertion; ERT, elastic resistance training; PTI, physical therapy intervention; Cal-EX, exercise plus Calanus oil; Pla-EX, exercise plus placebo.

##### 2.2.1.6 Exclusion criteria

Ineligible study types: Narrative reviews, systematic reviews, meta-analyses, conference abstracts, case reports, animal experiments, in-vitro experiments, and publications without complete extractable data were excluded.

Ineligible populations: Studies recruiting male participants, children, adolescents, young women, or women with unverifiable postmenopausal status were excluded. Studies focusing on patients in the acute phase of severe disease, where separate data for postmenopausal women could not be isolated, were also excluded.

Ineligible interventions: Acute single-bout exercise without multi-week chronic training intervention was excluded. Non-exercise interventions (e.g. pure nutritional supplementation, drug intervention without exercise component) were excluded. Yoga and tai chi were excluded given their mixed mind-body characteristics in this analysis.

Ineligible outcomes: Studies failing to report BDNF quantitative data were excluded, including those only reporting cognition, muscle strength, inflammatory markers or other indices without BDNF measurements. Studies from which BDNF data could not be extracted from text, tables or figures were also excluded.

Duplicate data: When multiple articles reported data from the same study population, only the publication with the most complete dataset, largest sample size, or best-matched outcome indicators was included to avoid double-counting participants.

Missing critical data: For studies lacking key information such as sample size, pre-post BDNF means, standard deviations or P-values, data were estimated from figures or converted via statistical methods where feasible. If meta-analyzable data still could not be obtained, such studies were only used for qualitative description or excluded.

Unclear intervention descriptions: Studies without explicit reporting of intervention duration, training frequency or basic training contents were excluded. Studies missing only intensity details but with complete remaining information were included and categorized as “intensity not reported or unclear” in subgroup or sensitivity analyses.

Prespecified subgroup rule: Only subgroups containing ≥3 independent original studies entered quantitative pooled meta-analysis. Subgroups with fewer than three studies were not pooled quantitatively and were only mentioned descriptively in discussion as exploratory observations.

### 2.3 Data extraction

All retrieved records were imported into NoteExpress 3.9 for literature management, and duplicate entries were removed. Subsequently, two reviewers independently performed two-stage screening: initial screening based on titles and abstracts, followed by full-text evaluation to assess eligibility against predefined inclusion criteria. Disagreements during screening were resolved by group discussion; when consensus could not be reached, a third reviewer made the final arbitration. The PRISMA-compliant flow diagram was plotted to visualize the whole procedure including retrieval, deduplication, exclusion, and final study inclusion.

After screening, two reviewers independently extracted relevant information from eligible studies using a pre-specified data-extraction form, followed by cross-checking to guarantee accuracy and consistency of extracted data. The main extracted items were as follows:

1. General information: first-author name, publication year, study country, journal title, study-design type, which described basic characteristics of included studies.
2. Participant characteristics: the exact original definition of postmenopausal status applied within each primary study; whether the sample contained mixed perimenopausal/postmenopausal participants or relied solely on age for classification, health conditions, age, sample sizes of experimental and control groups. For trials containing multiple intervention arms, sample size and corresponding intervention modality for each arm were recorded separately. Total unique participants for each study were documented separately from raw arm-level sample sizes.
3. Characteristics of structured exercise interventions:: intervention protocols for experimental and control groups, including training modality, intervention duration, training frequency, session duration, exercise intensity, sets, repetitions, and co-interventions. For combined-intervention studies, both resistance-training components and additional intervention contents were explicitly documented. All extracted interventions corresponded to multi-week chronic exercise programmes.
4. Outcome indicators: primary extracted outcomes comprised resting-state BDNF-related data, standard deviations, change values and P-values. For studies reporting medians and interquartile ranges, appropriate statistical approaches were adopted to convert data into means and standard deviations. If outcomes were only presented in figures, approximate numerical values were extracted via graphical estimation, and such estimation was annotated in corresponding tables.
5. Risk-of-bias information: items concerning random-sequence generation, allocation concealment, blinding implementation, incomplete outcome data, selective reporting, and other potential biases were extracted for subsequent methodological-quality assessment.

For data synthesis, extracted records were sorted according to participants, intervention modality, intervention duration, exercise intensity, and energy-supply pattern. Quantitative data were tabulated to facilitate comparisons of differential exercise modality effects on BDNF levels among postmenopausal women across individual studies. Studies unsuitable for quantitative pooling were synthesized narratively, with potential sources of heterogeneity interpreted. This data-extraction and organization procedure ensured transparency, reproducibility and reliability of the present meta-analysis results.

### 2.4 Statistical analysis

Statistical analyses were performed using Review Manager 5.4 software. The primary outcome of included studies was a continuous variable, namely peripheral-blood, serum, or plasma resting-state BDNF levels before and after intervention. Given potential discrepancies in BDNF specimen types, detection assays and units of measurement across individual studies, effect sizes were reported as standardized mean difference (SMD) with 95 % confidence interval (95% CI).

When means and standard deviations were directly reported in publications, these values were extracted for meta-analysis. Approximate numerical values were extracted from figures if results were only presented graphically. For trials with multiple intervention arms, arm-selection and shared-control-group handling followed rules defined in Section 2.2; similar intervention arms were pooled when necessary to avoid double-counting participants in control groups. Unique participant counts were kept separate from arm-level sample sizes to prevent over-counting individuals in total sample reporting.

Heterogeneity was assessed using the Q-test and I² statistic, with the significance threshold for the Q-test set at P < 0.10. The I² statistic quantified between-study heterogeneity. A fixed-effect model was adopted when I² ≤ 50 % and P ≥ 0.10, indicating low heterogeneity. A random-effect model was applied for pooled analysis when substantial heterogeneity was observed (I² > 50 % or P < 0.10).

Subgroup analyses were performed according to study characteristics, including exercise intervention modality, intervention duration, exercise intensity, population characteristics, and energy-supply pattern. Intervention modalities were categorized as resistance-training-dominant exercise, combined exercise training, vibration or vibration-combined training. Intervention durations were classified as 8 weeks, 12 weeks, and more than 12 weeks. Exercise intensity was stratified into low-intensity, moderate-intensity, and high-intensity subgroups.

Methodological quality of included studies was evaluated with the Cochrane Risk-of-Bias Tool. Assessment domains comprised random-sequence generation, allocation concealment, blinding of participants and personnel, blinding of outcome assessment, incomplete outcome data, selective reporting, and other sources of bias. Each domain was judged as low risk, unclear risk, or high risk of bias. We acknowledge important limitations when applying this tool for quasi-randomized and non-randomized studies; risk-of-bias judgements for non-RCT designs are interpreted cautiously and cannot be treated as equivalent to quality assessment tools specifically designed for non-randomized interventions.

Sensitivity analysis was carried out in Review Manager 5.4 to test the stability of pooled effect sizes by sequentially omitting one individual study at a time. Funnel plots and Egger’s test were applied to evaluate publication bias when a sufficient number of studies were available; descriptive assessment of publication bias was performed for small-sample sets. Two-tailed statistical tests were applied throughout, and P < 0.05 was defined as statistically significant.

## 3 Results

### 3.1 Study characteristics

Twelve RCT studies (including randomized controlled trials, quasi-randomized trials and clinical intervention studies) were included in the present meta-analysis; all included trials implemented multi-week chronic exercise interventions, and acute single-bout exercise research was excluded, involving 351 participants whose ages ranged from 51.37 to 84 years. For the primary meta-analysis, 173 participants were allocated to intervention groups and 178 to control groups after proportional splitting of shared control groups in multi-arm studies. **Table 1** reports for each study: study design type, original definition of postmenopausal status used in the primary article, and explicit marking for studies containing mixed or ambiguously defined postmenopausal samples. Tsai et al. (2025) was removed from the table. **Table 1** has been revised to match the final included literature. The included studies covered multiple structured exercise modalities, including resistance training, aerobic exercise, whole-body vibration training, inspiratory muscle training, dual-task training, and combined exercise protocols.

### 3.2 Study quality assessment

Risk-of-bias assessment for included studies was performed using the Cochrane Risk-of-Bias Tool. Seven domains were appraised via Review Manager 5.4 software: random-sequence generation, allocation concealment, blinding of participants and personnel, blinding of outcome assessment, incomplete outcome data, selective reporting, and other sources of bias (**Figure 2**).

**Figure 2:**
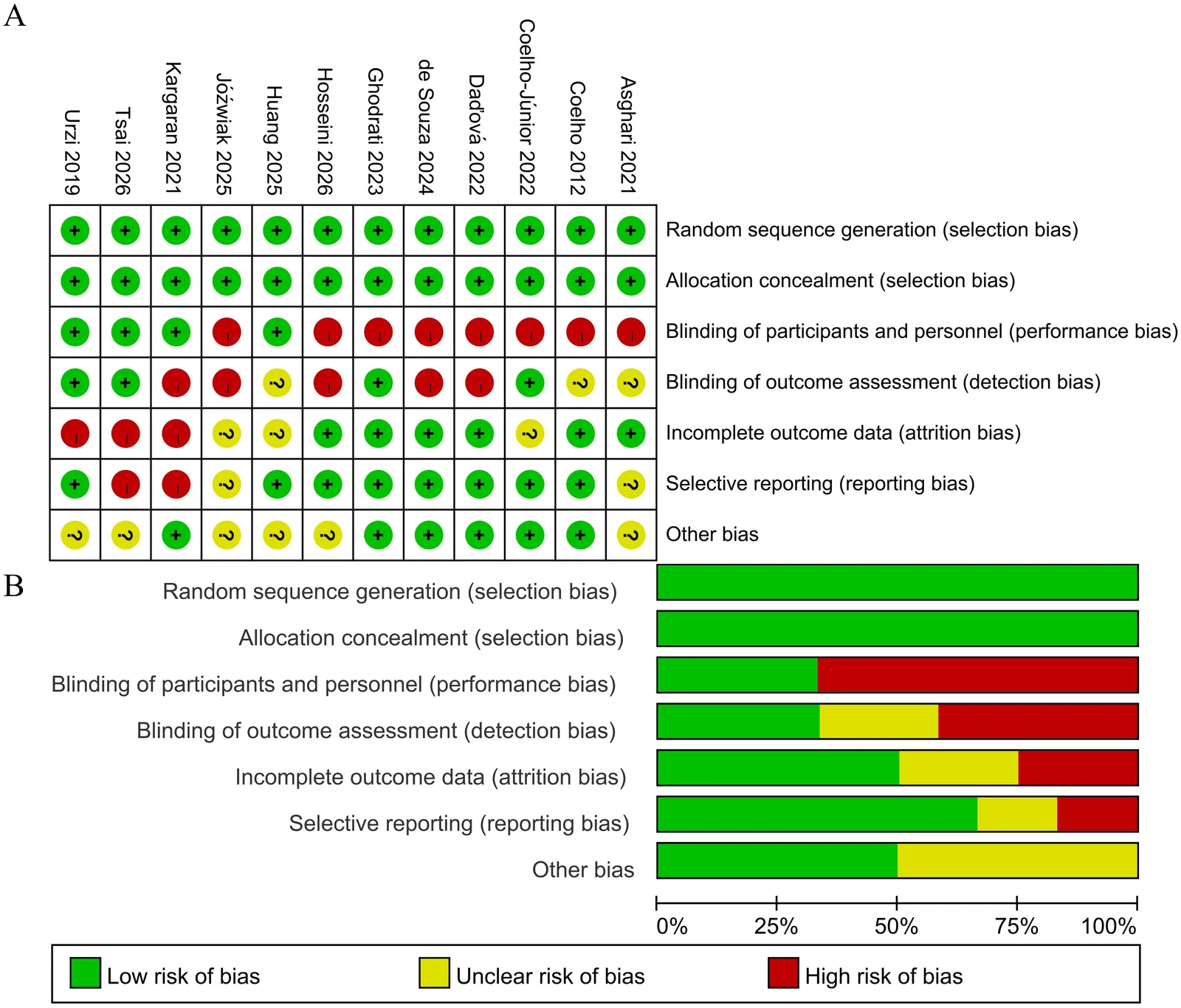
Methodological quality graph and summary of the included studies: **(A)** Risk of bias summary; **(B)** Risk of bias graph.

Twelve studies were included in the present meta-analysis. Not all studies were judged as low risk for random-sequence generation and allocation concealment. For quasi-randomized and non-randomized studies (Huang et al., 2025, Asghari et al., 2021, Coelho et al., 2012) that lacked explicit descriptions of random-sequence generation and allocation concealment, these two domains were rated as unclear risk of bias, in accordance with Cochrane RoB guidance. We explicitly note that Cochrane Risk-of-Bias is not purpose-built for non-randomized designs; conclusions from non-RCT studies are interpreted with extra caution due to this methodological limitation. For blinding of participants and personnel, eight studies were rated as high risk. This was primarily attributable to the inherent identifiability of interventions such as diverse structured exercise modalities, vibration training, stretching training, and no-exercise control, which prevented effective blinding of participants and trainers. Regarding blinding of outcome assessment, five studies were classified as low risk, three as unclear risk, and five as high risk, suggesting that several studies failed to adequately report whether laboratory or outcome assessors were blinded to group allocation.

For incomplete outcome data, six studies were assessed as low risk, four as unclear risk, and three as high risk, mainly due to insufficient reporting of participant dropout or imbalanced attrition between groups. With respect to selective reporting, eight studies were rated low risk, three unclear risk, and two high risk. For other sources of bias, seven studies were low risk and six were unclear risk, largely resulting from small sample sizes and inadequate reporting of baseline characteristics or intervention details.

Overall, the main sources of bias among the included studies were concentrated in blinding of participants and personnel and blinding of outcome assessment. Although BDNF was measured objectively via laboratory assays of serum or plasma specimens, variations across studies in blinding implementation, sample size, and data completeness may still exert influences on the stability of meta-analytic results.

### 3.3 BDNF

A total of 12 studies and 351 participants were included, comparing the effects of various chronic structured exercise interventions on the resting-state BDNF levels of postmenopausal women. After handling multi-arm trials and shared control groups per the prespecified rules, 173 participants were included in intervention groups and 178 in control groups for the primary pooled analysis, with no participant double-counted. No acute single-bout exercise data were incorporated in this pooled analysis. Due to differences in intervention methods, intervention duration, training intensity, population characteristics, and BDNF detection sample types among different studies, standardized mean difference (SMD) was used as the effect size (**Figure 3**).

**Figure 3:**
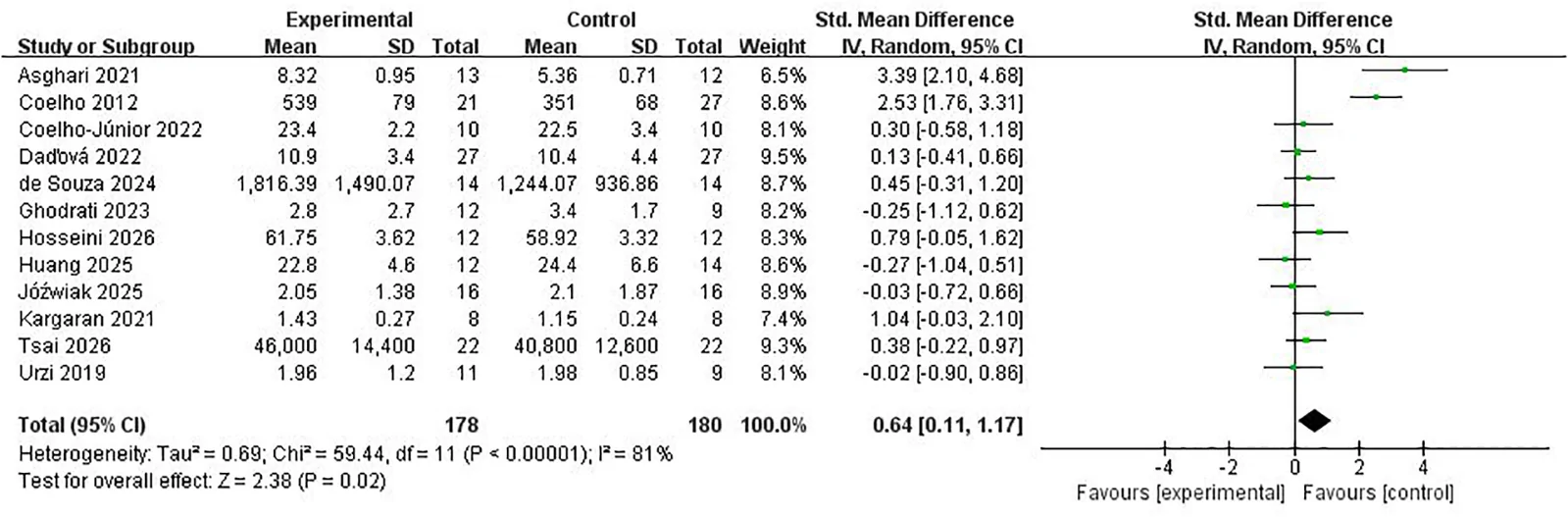
Effect of Exercise on BDNF.

The heterogeneity test results showed significant heterogeneity among the included studies (Tau² = 0.40, Chi² = 41.77, df = 11, P < 0.0001, I² = 74%). Since I² > 50% and P < 0.10, it indicates significant heterogeneity between studies, and thus a random effects model was used for the Meta-analysis.

The heterogeneity test results showed significant heterogeneity among the included studies (Tau² = 0.38, Chi² = 39.11, df = 10, P < 0.0001, I² = 72%). Since I² > 50% and P < 0.10, it indicates significant heterogeneity between studies, and thus a random effects model was used for the Meta-analysis.

The combined results showed that structured chronic exercise interventions significantly increased the resting-state BDNF levels of postmenopausal women, with a combined effect size of SMD = 0.52, 95% CI: 0.09-0.95, and the difference was statistically significant (Z = 2.34, P = 0.02). This result suggests that compared to the control group, general structured chronic exercise intervention may significantly increase the peripheral resting BDNF levels of postmenopausal women. The present pooled analysis only synthesised resting-state BDNF adaptations induced by multi-week chronic training, without mixing acute single-bout exercise-triggered transient BDNF responses.

### 3.4 Subgroup analysis

Subgroup analyses were performed in the present study to further explore potential exploratory candidate factors that may relate to heterogeneity regarding the effects of structured chronic exercise interventions on BDNF levels in postmenopausal women (**Table 2**). Only subgroups containing at least three independent studies were quantitatively pooled; small-sample strata with fewer than three studies were excluded from quantitative meta-analysis and are only discussed in the discussion section as preliminary exploratory clues. Stratification was conducted according to intervention modality, population characteristics, intervention duration, exercise intensity, and energy-supply pattern. Given the substantial between-study heterogeneity observed in the overall pooled analysis, it was necessary to investigate how methodological and population-level characteristics might correlate with summary effects. No formal statistical interaction tests between subgroups were performed; observed differences in significance across subgroups cannot be taken as definitive evidence of moderator effects or causal sources of heterogeneity.

**Table 2:** Subgroup analyses of exercise effects on BDNF concentrations in postmenopausal women.

Research Features	Subgroup standard	Study (sample)	SMD	95%CI	P	I2(%)	P (Heterogeneity)
Type of Exercise	Resistance-training-based exercise	7 (87)	1.07	0.12,2.01	0.03	87	< 0.00001
	Combined-exercise training	3 (55)	0.01	-0.37, 0.39	0.97	0	0.77
Demographic characteristics	Healthy Elderly/Female Postmenopausal	6 (82)	0.30	-0.05, 0.65	0.009	19	0.29
	Frailty/institutional-care residents	4 (73)	0.77	-0.35,1.88	0.18	90	< 0.00001
Intervention week classification	8 weeks	4 (54)	1.64	0.02,3.26	0.05	92	< 0.00001
	12 weeks	5 (65)	0.19	-0.71,0.55	0.29	0	0.41
	Over 12 weeks	3 (59)	0.25	-0.11,0.61	0.18	0	0.82
Strength classification	Medium intensity	9 (118)	0.45	-0.08-0.97	0.10	72	0.0003
Metabolic energy characteristics	Anaerobic Energy Supply Main	7 (87)	1.07	0.12,2.01	0.03	87	< 0.00001
	Mixed-energy-supply	4 (69)	0.10	-0.24,0.44	0.58	0	0.67

Subgroups containing fewer than three independent studies were not included in this table and were excluded from quantitative meta-analysis.

In the subgroup analysis for intervention modality, seven studies were included in the resistance-training-dominant subgroup, showing substantial heterogeneity (I² = 86 %), with a statistically significant pooled effect size (SMD = 1.04, 95 %CI: 0.10-1.98, P = 0.03). This finding indicated that resistance training significantly elevated BDNF levels among postmenopausal women. The combined-exercise-training subgroup comprised three studies with zero heterogeneity, yet the pooled effect was non-significant (SMD = 0.01, 95 %CI: −0.37-0.39, P = 0.97).

For population-characteristic subgroups, seven studies enrolled healthy older/postmenopausal women, with low heterogeneity (I² = 34 %) and a statistically-significant pooled effect (SMD = 0.41, 95 %CI: 0.04-0.77, P = 0.03). Subgroups for participants with obesity/metabolic abnormalities and frailty/institutionalized older adults exhibited high heterogeneity (I² = 95 % and I² = 90 %, respectively), and neither produced a statistically-significant pooled effect, suggesting that population health status constitutes an important source of heterogeneity.

In terms of intervention-duration subgroups, the 8-week subgroup presented a large effect size at the margin of statistical significance (SMD = 1.61, 95 %CI: 0.01-3.21, P = 0.05) accompanied by high heterogeneity (I² = 91 %). Heterogeneity was zero for both the 12-week and >12-week subgroups; nevertheless, their pooled effects failed to reach statistical significance. These exploratory results hint that short-term chronic exercise may be associated with larger effect estimates; this should not be interpreted as conclusive evidence of differing true effects.

Within exercise-intensity subgroups, only the moderate-intensity subgroup (9 studies) met the inclusion threshold for quantitative pooling, showing high heterogeneity (I² = 71 %), and nonsignificant pooled effect (SMD = 0.44, 95 %CI: −0.09-0.97, P = 0.10).

Regarding energy-supply-pattern subgroups, seven studies were assigned to the resistance-/anaerobic-energy-supply-dominant subgroup, which showed substantial heterogeneity (I² = 86 %) and a statistically-significant pooled effect (SMD = 1.04, 95 %CI: 0.10-1.98, P = 0.03). The aerobic-dominant subgroup had insufficient study numbers and was not quantitatively pooled; accordingly, we refrain from reporting meta-analytic conclusions about statistical significance for this under-powered stratum.

### 3.5 Sensitivity analysis

Sensitivity analysis was performed by sequentially omitting individual studies to evaluate the influence of each single trial on the pooled effect size and overall heterogeneity. The overall pooled analysis yielded a pooled effect size of SMD = 0.64(95 %CI: 0.11-1.17, P= 0.02), with substantial heterogeneity (I² = 81%). Full leave-one-out results for all 12 included studies are shown in **Table 3**. Each row corresponds to the pooled estimate obtained after removing the named study.

**Table 3:** Combined effects of BDNF after excluding individual studies.

Study	*SMD*	95% *CI*	*P* (Merge effect)	*I^2^*(%)
Asghari et al., 2021	0.45	-0.01, 0.90	0.05	85
Coelho et al., 2012	0.42	0.01, 0.84	0.05	77
Coelho-Júnior et al., 2020	0.67	0.10, 1.25	0.02	83
Daďová et al., 2022	0.70	0.11, 1.29	0.02	83
de Souza et al., 2024	0.66	0.08, 1.24	0.03	83
Ghodrati et al., 2023	0.72	0.16, 1.28	0.01	82
Hosseini et al., 2026	0.63	0.06, 1.20	0.03	83
Huang et al., 2025	0.73	0.17, 1.29	0.01	82
Jóźwiak et al., 2025	0.71	0.14, 1.28	0.02	82
Kargaran et al., 2021	0.61	0.05, 1.17	0.03	83
Tsai and Chang, 2026	0.67	0.08, 1.27	0.03	83
Urzi et al., 2019	0.70	0.14, 1.27	0.02	83
Overall	0.64	0.11, 1.17	0.02	81

Every row reports the pooled estimate obtained after removing the indicated study. The final row is the primary overall meta-analysis result with no study excluded.

After sequential exclusion of each included study, the pooled effect sizes fluctuated within the range of 0.42-0.73. All corresponding 95 % confidence intervals did not cross the line of no-effect, and all P-values remained less than 0.05, indicating persistent statistical significance of the pooled results. The I² statistic for heterogeneity varied from 77 % to 83 %. No removal of a single study resulted in a prominent disappearance of heterogeneity or reversal of the direction of pooled effect.

Accordingly, the sensitivity analysis demonstrated that individual studies exerted limited influence on the overall pooled estimate, and the present meta-analysis results were relatively stable. Nevertheless, heterogeneity remained substantial after omitting single studies, which suggested that heterogeneity might arise from multiple candidate factors, including intervention modality, training duration, exercise intensity, population characteristics, and specimen types for BDNF measurement.

### 3.6 Publication bias

Funnel plots were generated for each quantitatively pooled subgroup separately to assess potential publication bias. As illustrated in **Figure 4**, the funnel plots exhibited roughly symmetrical distribution. Egger’s test was further performed based on these funnel plots. The results showed that no statistically significant asymmetry was detected via Egger’s test. However, given the small number of included studies (n = 13 overall), the statistical power of Egger’s test and funnel-plot judgement is low; therefore, we cannot definitively rule out publication bias.

**Figure 4:**
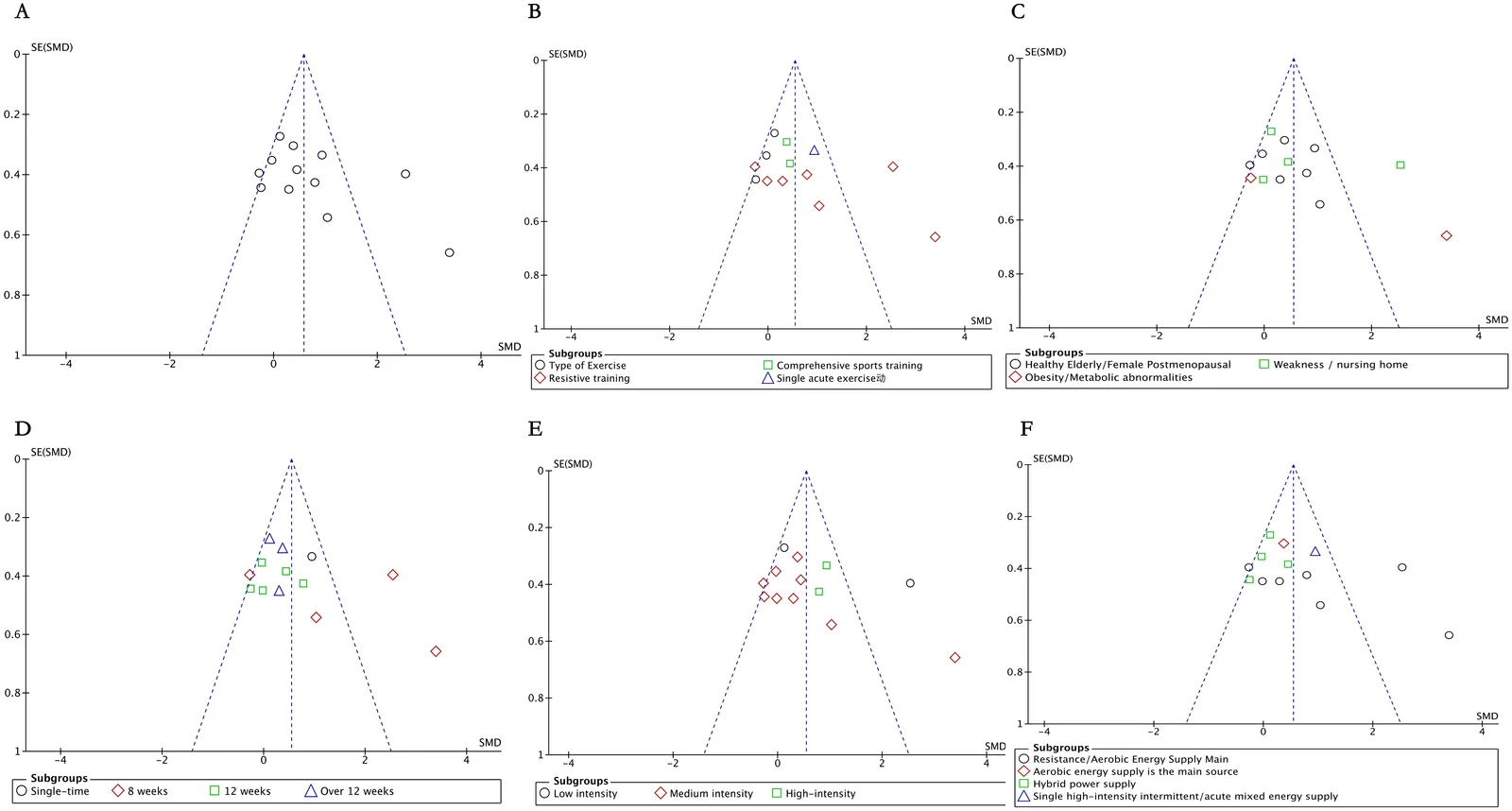
Funnel plots of BDNF: **(A)** combine funnel chart; **(B)** type of exercise; **(C)** demographic characteristics; **(D)** intervention week classification; **(E)** strength classification; **(F)** intervention on exercise metabolic energy characteristics.

## 4 Discussion

### 4.1 Effects of resistance training on BDNF in postmenopausal women

Physical training serves as an important non-pharmacological strategy to modulate brain-derived neurotrophic factor (BDNF). BDNF is primarily involved in neuronal survival, synaptic plasticity, learning-memory processes and emotional regulation. Changes in its circulating concentration are closely linked to brain health and cognitive function (Leal et al., 2017; Miranda et al., 2019). Previous meta-analyses have demonstrated that exercise training generally elevates peripheral resting BDNF levels, yet discrepancies exist across exercise modalities, population subgroups and training protocols. Sufficient evidence has been accumulated for aerobic exercise, whereas the comparative efficacy of different structured exercise modalities on BDNF regulation remains controversial (Szuhany et al., 2015; Dinoff et al., 2016). Focusing specifically on postmenopausal women, the present meta-analysis revealed that resistance-training-related chronic interventions significantly increased peripheral resting BDNF levels compared with control conditions (SMD = 0.52, 95 %CI: 0.09-0.95, P = 0.02).

Postmenopausal women represent a population of particular interest for BDNF-oriented research. Ovarian decline and marked oestrogen reduction after menopause trigger multi-system alterations in neuroendocrine activity, metabolism, inflammatory status and cognitive performance. Brinton et al. (2015) proposed that the pre-menopausal phase acts as a critical transitional window for female neural function; oestrogen withdrawal may impair cerebral energy metabolism, synaptic function and neuroprotective capacity. Prior investigations have confirmed reciprocal crosstalk between oestrogen and BDNF signalling. Oestrogen modulates BDNF expression and its receptor-mediated pathways to regulate neuroplasticity and cognition (Luine and Frankfurt, 2013).

Accordingly, postmenopausal BDNF fluctuations are potentially associated with cognitive decline, mood disturbance and attenuated neuroprotection among women. Our finding that chronic resistance training elevates resting-state BDNF levels in postmenopausal women suggests resistance training may partially counteract the decline in neurotrophic support following menopause. It should be emphasized that the present meta-analysis measured only peripheral circulating resting-state BDNF. Peripheral BDNF elevation cannot be directly interpreted as evidence of improved central neuroplasticity or clinical brain-health outcomes in postmenopausal women.

Subgroup analysis further identified modality-specific differences: resistance-training-dominant exercise yielded the most prominent BDNF-enhancing effect, while combined exercise training showed no significant efficacy. The BDNF-enhancing effect of resistance-dominant exercise may be explained by skeletal-muscle contraction-derived stimulation and regulation via the muscle-brain axis. Robust muscular contraction and motor-unit recruitment during resistance training facilitate the release of myokines such as irisin and insulin-like growth factor-1 (IGF-1). These humoral factors enter systemic circulation and target the central nervous system to promote BDNF expression. Wrann et al. (2013) reported that physical activity upregulates hippocampal BDNF through the PGC-1α/FNDC5 signalling cascade, providing mechanistic evidence for skeletal-muscle-originated regulation of neurotrophic factors. Moreover, resistance training improves muscular strength, physical performance, insulin sensitivity and chronic inflammatory profiles. These physiological adaptations collectively construct a favourable internal milieu for BDNF elevation (Fragala et al., 2019).

Despite the significant pooled effect of chronic exercise on resting-state BDNF, substantial between-study heterogeneity was observed (I² = 72 %, P < 0.0001). This indicates that the magnitude of BDNF responses to exercise is not consistent across trials and can be modulated by intervention modality, training duration, exercise intensity, participant health status, BDNF specimen matrix and blood-sampling time-points. Earlier studies also highlighted that peripheral BDNF is highly sensitive to exercise type, exercise dosage, sampling timing and individual baseline conditions (Szuhany et al., 2015; Dinoff et al., 2016). Therefore, subgroup analyses were performed in the current meta-analysis only for strata containing ≥3 independent studies to identify potential moderators governing BDNF responses to chronic exercise in postmenopausal women and to inform future exercise-prescription development. Several theoretically-interesting moderators (e.g. high-intensity training) could not be quantitatively pooled because fewer than three relevant studies were available; these factors are only discussed as exploratory hints.

### 4.2 Moderating factors for BDNF responses induced by resistance training

Most studies investigating the relationship between exercise intervention and BDNF indicate that BDNF responses are affected by multiple factors, including exercise modality, exercise intensity, training duration, participant age and health status. Meta-analyses by Dinoff et al. (2016)and Szuhany et al. (2015) both reported that exercise generally increases peripheral resting BDNF levels, but considerable heterogeneity exists across studies, suggesting that exercise-induced BDNF alterations are not determined by a single factor. The overall pooled analysis of the present study showed that resistance training and related chronic interventions significantly elevated resting-state BDNF levels in postmenopausal women, with high heterogeneity (I² = 72 %, P < 0.0001). Therefore, subgroup analyses were performed from five perspectives: intervention modality, population characteristics, intervention duration, exercise intensity, and energy-supply pattern, to interpret sources of heterogeneity and explore possible mechanisms by which resistance training improves BDNF levels in postmenopausal women.

#### 4.2.1 Intervention modality

A core novelty of this meta-analysis is the comprehensive inclusion of multiple chronic structured exercise modalities, while excluding transient acute single-bout exercise trials. Quantitative pooling confirmed that resistance-training-dominant exercise exerts a significant BDNF-promoting effect, while combined exercise training showed no statistical efficacy. Vibration-combined exercise lacked sufficient study samples for quantitative synthesis, and their effects remain to be verified by accumulating more primary trials. These exploratory subgroup observations suggest exercise modality may correlate with resting-state BDNF responses; we cannot draw definitive causal conclusions about superior regulatory efficacy of resistance-based exercise relative to other exercise forms in absence of formal subgroup interaction testing.

#### 4.2.2 Population characteristics

Existing studies have indicated that age, oestrogen level, metabolic status and baseline inflammatory levels may influence BDNF expression as well as BDNF responsiveness to exercise stimuli. The perimenopausal and postmenopausal periods are important phases for neuroendocrine and cerebral metabolic alterations in women. Oestrogen decline can affect synaptic plasticity, cerebral energy metabolism and neuroprotective functions (Wrann et al., 2013; Brinton et al., 2015). In the present study, the subgroup of healthy older/postmenopausal women presented a significant increase in BDNF (SMD = 0.41, 95 %CI: 0.04-0.77, P = 0.03) with low heterogeneity (I² = 34 %). This finding suggests that postmenopausal women with relatively stable health status yield more consistent resting-state BDNF responses to structured chronic exercise intervention. The subgroup of participants with obesity or metabolic abnormalities contained only two studies and was excluded from quantitative pooling; whether metabolic status modulates BDNF response needs more evidence.

#### 4.2.3 Intervention duration

Exercise-related BDNF responses can be categorized into transient acute responses and long-term chronic adaptations. BDNF concentrations rise shortly after a single bout of acute exercise, whereas resting-state BDNF changes induced by long-term training are susceptible to training protocols, blood-sampling timepoints and individual adaptive status. Only chronic training-related resting-state BDNF data were synthesized in this meta-analysis. The 8-week subgroup presented a large effect size at borderline statistical significance. By contrast, subgroups of 12 weeks and longer-than-12-week interventions failed to reach statistical significance, these exploratory findings hint that resting BDNF may not keep increasing persistently following chronic training, but this observation requires further independent validation. Potential explanations include physiological adaptation to regular training, insufficient progressive overload, inconsistent blood-sampling schedules, and the relatively short half-life of circulating BDNF. Future research should discriminate BDNF fluctuations immediately post-exercise from resting-state BDNF alterations after chronic training to improve the interpretability of findings.

#### 4.2.4 Exercise intensity

Exercise intensity represents a critical training parameter modulating BDNF responses. Previous evidence suggests that higher-intensity exercise provokes more pronounced metabolic stress, lactate accumulation and neuroendocrine activation, which are correlated with enhanced BDNF expression (Leal et al., 2017; Tsai and Chang, 2026). High-intensity and low-intensity subgroups contained fewer than three studies each and were therefore excluded from quantitative meta-analysis; we cannot draw meta-analytic conclusions regarding the comparative effectiveness of high-intensity chronic exercise. Observations from individual primary studies provide preliminary mechanistic hints: high-intensity exercise may trigger recruitment of high-threshold motor units, augmented muscular tension, and greater release of lactate and myokines. El Hayek et al. (2019) reported that exercise-derived lactate activates hippocampal BDNF signalling through a SIRT1-dependent pathway, providing mechanistic support for high-intensity-exercise-driven BDNF upregulation. For postmenopausal women, high-intensity exercise should be prescribed individually with full consideration of bone mineral density, joint function, chronic-disease status and prior exercise experience. We emphasize that such clinical prescription guidance is based on mechanistic reasoning and individual-study evidence rather than pooled meta-analytic subgroup results.

#### 4.2.5 Energy-supply pattern

Exercise-induced BDNF alterations can be interpreted from the perspective of energy metabolism. Aerobic exercise modulates BDNF by improving cardiorespiratory fitness, cerebral blood flow and metabolic health. In contrast, resistance-dominated or anaerobic-predominant chronic exercise may stimulate BDNF release via forceful muscular contraction, lactate accumulation and enhanced metabolic stress (Schiffer et al., 2011; Szuhany et al., 2015). In the present meta-analysis, the resistance/anaerobic-dominant subgroup yielded a significant increase in BDNF (SMD = 1.04, 95 %CI: 0.10-1.98, P = 0.03). This exploratory pooled result is limited by lack of formal subgroup interaction testing and high overlap between resistance-training-based and anaerobic-predominant study samples; it cannot confirm anaerobic-predominant exercise produces definitively superior effects. This finding suggests that exercise characterized by resistance and anaerobic metabolic stimulation may be preferable for triggering resting-state BDNF elevation among postmenopausal women. Such training produces pronounced muscular tension, neuromuscular activation and lactate-related signalling, thereby regulating BDNF expression through peripheral-to-central pathways. Collectively, energy-supply pattern may partially correlate with resistance-training-evoked BDNF responses by modulating metabolic stress magnitude and muscle-to-brain signalling transduction (Fragala et al., 2019). This energy-supply-pattern classification was predefined *a priori* according to exercise-physiological criteria before quantitative analysis. We observed that the resistance/anaerobic-energy-supply-predominant subgroup yielded identical pooled estimates to the resistance-training-based exercise subgroup, which indicates high overlap between these two categories of study samples. This overlap is acknowledged as one limitation of this subgroup stratification. The aerobic-dominant subgroup had insufficient study numbers and was not quantitatively pooled.

### 4.3 Study limitations

Several limitations of this meta-analysis should be acknowledged. First, the number of included studies (n = 12) and overall sample size were relatively small; many potential moderators could not be quantitatively analysed because corresponding subgroups contained fewer than three primary studies, which limits our ability to disentangle sources of heterogeneity. This meta-analysis deliberately excluded acute single-bout exercise trials and only synthesized evidence from multi-week chronic exercise interventions; therefore, our conclusions are only applicable to chronic training-induced resting-state BDNF changes and cannot be generalized to transient BDNF responses after one-off acute exercise. Second, substantial variability existed across primary studies with respect to exercise intervention modalities, training duration, exercise intensity, participants’ health conditions, BDNF specimen matrix and blood-sampling time-points, leading to high overall heterogeneity. Third, eligible original studies focusing purely on single exercise modality among strictly-defined postmenopausal women are scarce. Therefore we included multiple forms of structured chronic exercise interventions, and only performed quantitative subgroup analysis for strata with ≥3 studies. Fourth, part of the included studies mixed perimenopausal and postmenopausal participants or only used age to infer postmenopausal status; restricted sensitivity analyses were performed to mitigate this limitation. Fifth, formal subgroup-difference interaction tests could not be reliably conducted even for some eligible subgroups due to limited sample size. Sixth, the original Cochrane Risk-of-Bias tool was applied instead of RoB 2, because several included studies were quasi-randomized or non-RCT clinical intervention studies. Seventh, assessment of publication bias was hampered by limited study count (n = 12), and publication bias could not be fully excluded.

Based on current findings, priorities for future investigations are proposed as follows: (1) Standardize structured chronic exercise intervention protocols and explicitly report training modality, intensity, frequency, sets, repetitions, rest intervals and principles of progressive overload to reduce inter-study heterogeneity. (2) Unify BDNF specimen types, laboratory detection methods and blood-sampling schedules; strictly discriminate acute post-exercise transient BDNF fluctuations from resting-state BDNF adaptations following chronic training. More primary studies are needed for high-intensity exercise and special-population subgroups to reach ≥3 studies for reliable subgroup meta-analysis. Independent meta-analyses focused on acute single-bout exercise are also required to explore acute-phase BDNF responses. (3) Enlarge sample sizes and explicitly report clear diagnostic definitions for postmenopausal status; recruit pure postmenopausal-only participants and avoid mixing perimenopausal and postmenopausal groups without separate-group data output, to improve external validity. (4) Conduct long-term follow-up studies to evaluate sustained influences of chronic exercise on BDNF, cognitive function, mood status, physical performance and intervention safety. Moreover, mechanistic research should incorporate measurements of lactate, irisin, IGF-1, inflammatory mediators, oestrogen levels and cognitive outcomes to elucidate the physiological pathways whereby different exercise modalities modulate circulating resting-state BDNF. Overall, more rigorously-designed, transparently-reported and adequately-followed-up high-quality randomized controlled trials are required to define optimal exercise prescriptions for improving BDNF status and neurological health in postmenopausal women.

## 5 Conclusion

General structured chronic exercise interventions significantly elevate peripheral resting-state BDNF levels among postmenopausal women, indicating their potential value for enhancing neuroplasticity and cerebral health in this population. Among diverse chronic exercise regimens, structured exercise protocols dominated by resistance stimuli, high intensity, and anaerobic energy metabolism exert more prominent and stable BDNF-upregulating effects in healthy postmenopausal women. The present meta-analysis only synthesised evidence of multi-week chronic training and does not cover acute single-bout exercise effects. This study confirms the neuroprotective potential of chronic exercise for postmenopausal women and highlights exercise modality and metabolic characteristics as key considerations for individualized brain health-oriented exercise prescription. Further high-quality research is warranted to clarify optimal training regimens and standardized measurement criteria for exercise-induced BDNF modulation in this specific population.

## Data Availability

The original contributions presented in the study are included in the article/supplementary material. Further inquiries can be directed to the corresponding author.
